# Vocal learning in songbirds: the role of syllable order in song recognition

**DOI:** 10.1098/rstb.2020.0248

**Published:** 2021-10-25

**Authors:** Carien Mol, Johan J. Bolhuis, Sanne Moorman

**Affiliations:** ^1^ Cognitive Neurobiology and Helmholtz Institute, Department of Psychology, Utrecht University, PO Box 80086, 3508 TB Utrecht, The Netherlands; ^2^ Department of Psychology, University of Cambridge, Cambridge, UK

**Keywords:** songbirds, syllable sequence, song recognition, phonotaxis, auditory song memory, tutor song

## Abstract

Songbird vocal learning has interesting behavioural and neural parallels with speech acquisition in human infants. Zebra finch males sing one unique song that they imitate from conspecific males, and both sexes learn to recognize their father's song. Although males copy the stereotyped syllable sequence of their father's song, the role of sequential information in recognition remains unclear. Here, we investigated father's song recognition after changing the serial order of syllables (switching the middle syllables, first and last syllables, or playing all syllables in inverse order). Behavioural approach and call responses of adult male and female zebra finches to their father's versus unfamiliar songs in playback tests demonstrated significant recognition of father's song with all syllable-order manipulations. We then measured behavioural responses to normal versus inversed-order father's song. In line with our first results, the subjects did not differentiate between the two. Interestingly, when males' strength of song learning was taken into account, we found a significant correlation between song imitation scores and the approach responses to the father's song. These findings suggest that syllable sequence is not essential for recognition of father's song in zebra finches, but that it does affect responsiveness of males in proportion to the strength of vocal learning.

This article is part of the theme issue ‘Vocal learning in animals and humans’.

## 1. Introduction

Vocal imitation learning is a rare trait in the animal kingdom, but is common among songbirds [1]. Songbirds learn to adjust their vocalizations to match an auditory model, and there are many behavioural, neural and genetic similarities with speech acquisition in human infants [2–4]. The zebra finch (*Taeniopygia guttata*) is a widely used animal species to study the biological basis of vocal learning. Only zebra finch males sing, and they learn their song from adult males, including their father. Female zebra finches do not produce learned vocalizations, but they also form a memory of their father's song [5].

**Figure 1.**
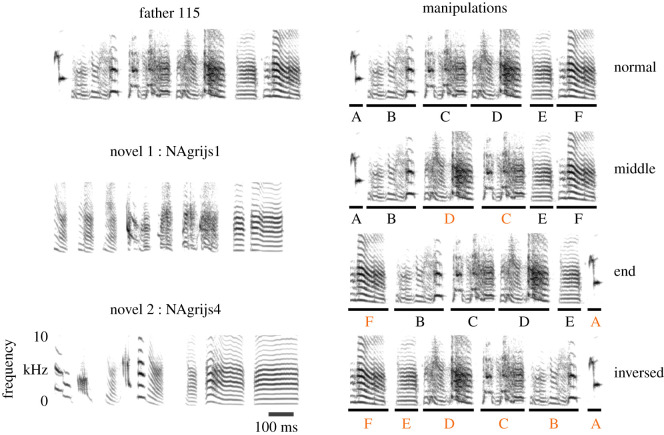
Stimulus design. Left: spectrograms of a representative motif of one of the fathers in our experiment (top; see the electronic supplementary material, audio files) and corresponding novel songs (middle and bottom). Scale bars at the bottom left are for all spectrograms. Right: the four different manipulations of the father's motif; lines and capital letters under the motif indicate the syllables, separated by silences. The orange colour of the letters specifies syllables with an altered position. Novel song stimuli received the same order manipulations (not shown). All spectrograms were created using Praat software.

Zebra finch song is characterized by a linear structure with a stereotyped ordering of syllables, the smallest units of song that are separated by a brief silent pause (figure 1). However, the importance of sequential information for perception and recognition remains unclear. It is not known if and how birds segment or parse song into smaller acoustic units, how these are memorized, and eventually used to recognize songs in their natural habitat (see for a review [6]). There is evidence to suggest that the order of zebra finch song syllables may follow certain rules, e.g. call-like harmonic stacks are likely to go at the end of the song [7].

Perceptually, zebra finches can hear changes in the order of syllables, even subtle ones. Previous studies demonstrated that zebra finches are able to discriminate between very similar songs, that only differ in the position of some song elements [8–10]. However, zebra finches may not always pay much attention to the ordering of syllables [11]. Zebra finch females did not show a preference for normal father's song over father's song in which two middle syllables had been switched [12]. In operant tasks, zebra finches responded strongly to unfamiliar, conspecific song stimuli with changes in local acoustic structure but much less to stimuli with changes in syllable ordering [9,10,13]. Interestingly, when familiar songs were used as stimuli, sensitivity to changes in syllable-order improved significantly [13].

Here, we investigated whether syllable order contributes to auditory recognition of learned father's song in male and female zebra finches that were raised in single-family cages, i.e. a setting with one available adult model. We tested father's song recognition using playback experiments with father's song and unfamiliar song as stimuli. Previously in such experiments, zebra finches displayed differential behavioural responses towards father's versus unfamiliar song, demonstrating recognition of the father's song (e.g. [5,12,14]). In particular, we investigated the importance of the sequential order of song syllables for song recognition in zebra finches by order-manipulating learned father's song. The order of syllables was altered in four possible different ways, ranging from small changes that may not attenuate recognition [12], to a complete inversion of syllable order that we hypothesized may significantly affect recognition.

## 2. Methods

### (a) Experimental subjects

All procedures were performed in compliance with European law and approved by the Animal Experiments Committee of Utrecht University. Adult zebra finch males (*n* = 17, mean age = 504 d, range = 292–1096 d) and females (*n* = 15, mean age = 399 d, range = 289–518 d) were bred in the Central Animal Facility of Utrecht University. Subjects and their siblings were raised by their father and mother until 70 days after hatching, to ensure ample opportunity to form a memory of the father's song. The male subjects also imitated the father's song. We determined the percentage of a father's song motif that was copied by the adult male subjects, as an indication for the strength of song imitation (using the %similarity score from Sound Analysis Pro [15]; see the electronic supplementary material). Although the subjects sometimes did not imitate all father's syllables and/or added novel sounds to their songs, we found that none of the imitated syllables occurred in a shuffled order compared to the father's song (e.g. electronic supplementary material, figure S1). In total, nine different fathers were used; some subjects shared the same parents (see the electronic supplementary material, table S1). After day 70, birds were housed in single-sex aviaries in the same room. The birds were maintained on a 15 : 9 h on : off light schedule and provided with water and food ad libitum.

### (b) Stimulus design

A full zebra finch song sequence is called a song bout [16], and it consists of repetitions of what we call the ‘motif’. A motif is a stereotyped, unique song that consists of song units which we call ‘syllables’, separated by silences (figure 1). Even though syllables can sometimes be composed of two or more different subparts, we only call them ‘syllables’ if they are separated by a silence of at least 10 ms. For the song stimuli, we used 15 or 16 motifs per bird, and used Audacity v. 1.3.13-beta and Praat v. 5.3.83 to create the stimuli (see the electronic supplementary material).

### (c) Experimental design

Subjects’ father's song recognition was examined using a laboratory playback experiment in which we measured the behavioural responses of birds towards two alternating stimuli (see the electronic supplementary material, figures S2 and S3). A difference in behaviour directed towards the familiar versus unfamiliar stimulus implies recognition of the familiar stimulus. The apparatus and stimulus playback design are comparable to previously described ‘phonotaxis preference tests' (e.g. [14]; see the electronic supplementary material, figure S2). In addition to the location of the bird in relation to the auditory stimuli (phonotaxis), we analysed vocal responses to the stimuli; songs and loud calls (call assay). These both are spontaneous, natural behavioural responses of the zebra finches that occur during acoustic playbacks without any prior training. Moreover, we used biologically relevant stimuli (which can be important: [6,13]), namely unfamiliar conspecific (novel) songs, and the learned father's song.

We conducted five playback tests. In four of these, we manipulated the syllable order in both the father's and novel stimuli (figure 1): normal sequence of syllables (normal), as a control test; the middle two syllables switched (middle); the first and last syllables switched (end) or an inversed order of the song syllable sequence (inversed). In order to obtain the different syllable sequences, we cut between all syllables in the silence interval (just before the onset of the next syllable) and pasted them back together in the intended order, using a crossfade of 0.5 ms to prevent cutting artefacts. These manipulations were applied to both the father's and novel songs. In the fifth test, we directly compared the normal father's song with the inversed father's song (see the electronic supplementary material, table S2 for an overview of the five tests).

### (d) Behavioural analyses

Two observers who were blind to the conditions of the test, each watched and listened to half of the videos, and scored the behaviours of the birds using JWatcher-Video v. 1.0. We added ‘observer’ as a control variable to the linear mixed-effects models (see ‘Statistical analyses’ below), but found no main effects of observer (all n.s., *p* > 0.4), suggesting that the two observers did not significantly differ in their scoring method. The scored behaviours of the birds were divided into two categories (cf. [16]): approach zone duration (time the bird spent on the left, middle and right zone of the apparatus) and vocal behaviour (number of loud calls in males and females and song bouts in males). We calculated within-bird difference scores of the behavioural responses towards father's minus novel stimulus to analyse whether the birds differentiated between the two stimuli (see the electronic supplementary material).

### (e) Statistical analyses

We used linear mixed-effects models to compare the birds' behavioural responses across experimental conditions, with subject as a random variable, and sex and sequence (normal, middle, end, inversed) as predictor variables. Furthermore, we explored whether adding similarity (%similarity score with the father's song) to the model explained some of the male subjects' behavioural responses. Likelihood ratio tests were used to determine the significant main effects of each predictor, in which case the associated model parameters were further evaluated with *t*-tests (via Satterthwaite's degrees of freedom method). The intercept of the model showed whether the mean difference score significantly deviated from zero, and is therefore used as a measure of father's song recognition. After model selection, assumptions were verified by visually inspecting plots of residuals for having a normal distribution and constant variance.

**Figure 2.**
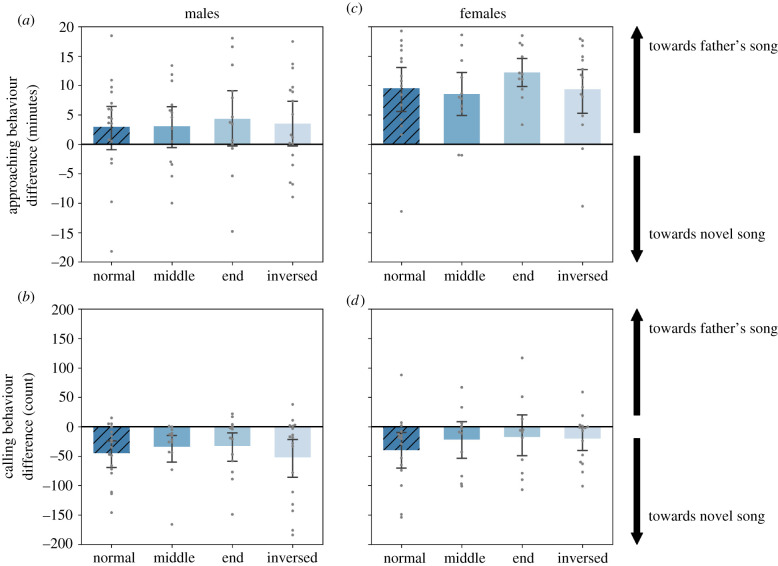
Birds responded differentially to father's song in all order-manipulated tests. Difference scores (father's—novel, see methods) show that subjects spent more time on the side at which father's song was played (*a*,*c*) and called more in response to the novel song than father's song (*b*,*d*). We found no differences between the order-manipulated tests, indicating recognition of the father's song in all sequence orders. Bars indicate the mean, whiskers the 95% error bars and data points the individual scores (left, males, *n* = 13–17; right, females, *n* = 11–15). Hatched bars indicate the unchanged syllable order (normal). See main text for statistics.

**Figure 3.**
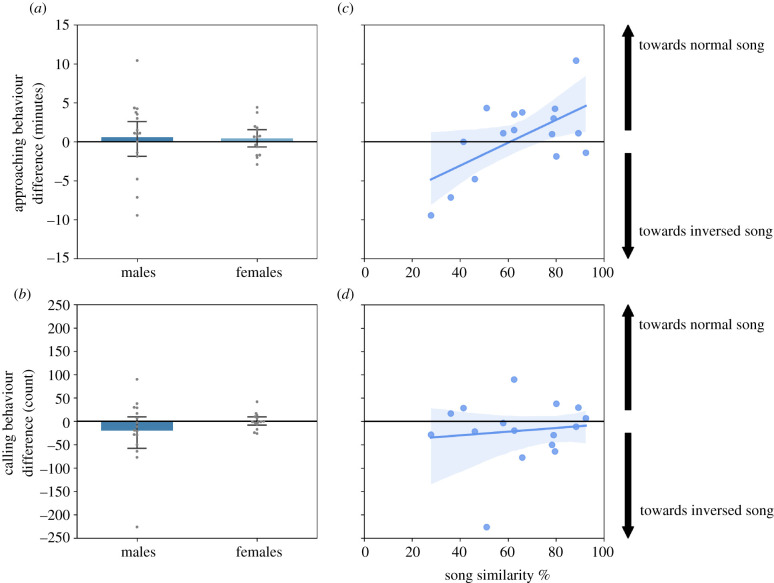
Normal father's versus the inversed father's song. Left: mean difference scores show that male and female subjects approached (*a*) and called (*b*) about equally in response to the normal and inversed song (means are not significantly different from zero; whiskers indicate 95% error bars). Right: the linear regression lines (with 95% confidence intervals) visualize the relationship with the strength of father's song imitation in males, demonstrating a significant correlation between %similarity and approaching behaviour (*c*), but not calling behaviour (*d*). See main text for statistics.

In addition, we used one-sample *t*-tests to investigate whether the birds recognized their father's song within the ‘normal’ sequence test (figure 2, hatched bars) and the ‘normal-inversed’ test (figure 3*a,b*). In the latter test, we also calculated Pearson's correlations between the behavioural difference scores and male subjects' song similarities to their father's songs (figure 3*c,d*).

Data analyses (see the electronic supplementary material) were performed in Python v. 3.6.12 (packages SciPy v. 1.5.2, seaborn v. 0.11.0, pandas v. 1.0.5), with the exception of the linear mixed-effects models, which were performed in R (v. 3.6.1, function *lmer* from the lmerTest-3.1–2 package).

## 3. Results

### (a) Recognition of father's song in males and females

In the normal sequence (father's versus novel songs with normal syllable order), both male and female zebra finches spent more time on the side of the apparatus where the father's song was played (figure 2*a,c*, hatched bars; see also the electronic supplementary material, figure S4 for raw data), and produced more loud calls in response to the novel song (figure 2*b,d*, hatched bars, see also the electronic supplementary material, figure S5 for raw data). In females, this was significant in both their calling behaviour (*t*_15_ = −2.49, *p* = 0.026) as well as in their approaching behaviour (*t*_15_ = 4.64, *p* < 0.001). In males, this was only significant in their calling (*t*_17_ = −3.96, *p* = 0.001), and not in their approaching behaviour (*t*_17_ = 1.46, *p* = 0.162). Thus, both sexes showed a differential response towards the father's song compared to the novel song in at least one behavioural measurement, indicating recognition of the song they had heard early in life.

### (b) Zebra finches recognized father's song when syllable order was altered

We found no significant effects of sequence manipulations on the difference scores of approaching behaviour (figure 2*a,c*; sequence: *χ*^2^_3_ = 2.49, *p* = 0.477, sex*sequence: *χ*^2^_3_ = 0.75, *p* = 0.861) nor calling behaviour (figure 2*b,d*; sequence: *χ*^2^_3_ = 1.54, *p* = 0.673, sex*sequence: *χ*^2^_3_ = 2.38, *p* = 0.498). This indicates that the birds differentiated between the father's and novel song in a similar manner in all four tests. Furthermore, a comparison of males and females revealed a significant main effect of sex on the approach zone difference scores (*χ*^2^_1_ = 9.62, *p* = 0.002), in which females spent more time than males on the side at which the father's stimulus was played back (females–males: *b* = 6.56, *t*_33_ = 3.33, *p* = 0.002). By contrast, there was no significant main effect of sex on call responses (*χ*^2^_1_ = 1.46, *p* = 0.226). In male subjects, we also analysed the number of song bouts produced in response to the father's and novel song stimuli showing similar results to those on calling behaviour (see the electronic supplementary material, figure S6).

Evaluation of the models showed that the overall father's versus novel song differentiation is significant in both approaching behaviour (intercept > 0; figure 2*a,c*; males: *b* = 3.35, *t*_33_ = 2.49, *p* = 0.018, females: *b* = 9.91, *t*_33_ = 6.90, *p* ≤ 0.001), as well as calling behaviour (intercept < 0; figure 2*b,d*; *b*= − 37.17, *t*_31_ = − 4.50, *p* < 0.001). Thus, the birds showed differential behaviour in the tests with the normal-order and all the order-manipulated stimuli, spending more time at the father's side and calling and singing more in response to the novel stimuli, demonstrating recognition of the father's song regardless of syllable order.

### (c) Behavioural responses to order-manipulated songs may be related to the strength of song imitation

Next, we explored whether song similarity to the father's song could explain some of the male subjects' behavioural responses. To this extent, we added similarity to the linear mixed-effects model and found a significant main effect of similarity on the difference scores of the approaching behaviour (similarity: *χ*^2^_1_ = 4.49, *p* = 0.034). Males with worse copies of the father's song tended to show a stronger approaching response to the father's song than subjects with better copies (similarity: *b* = −0.14, *t*_17_ = −2.28, *p* = 0.036), and there was some indication that the relationship between the strength of song imitation and the approach responses was stronger in the more substantial sequence changes (end, inversed; see the electronic supplementary material, figure S7). However, in the associated mixed model analysis, we found no interaction effect with sequence (similarity*sequence: *χ*^2^_6_ = 9.65, *p* = 0.140), and thus no post hoc analyses were done for the individual order-manipulated tests. For calling behaviour, we found no relationship to the strength of song imitation (all *p* > 0.05). In conclusion, our results suggest that the birds recognized the father's song, regardless of syllable-order manipulations, but that in males, approach responses to order-manipulated songs may be related to the strength of song imitation.

### (d) Birds had no preference for normal-order over inversed-order father's songs

Lastly, we directly contrasted normal-order father's song and inversed-order father's song. In this test, the approach zone and loud calls difference scores for both males and females were not significantly different from zero (all *p* > 0.05; figure 3*a,b*; see also the electronic supplementary material, figures S4 and S5 for raw data). Thus, at the group level, both male and female zebra finches behaved similarly towards normal and inversed father's song. This finding confirms the results from the first four playback tests, namely that birds recognized the father's song after the sequential order of syllables had been inversed.

However, when we compared the male subjects' individual level of song imitation to approach zone behaviour, we found that that the strength of song imitation was correlated significantly with the approach responses (Pearson's *r* = 0.61, *p* = 0.012; figure 3*c*). This was not true for calling behaviour (figure 3*d*, n.s.). Thus, although birds had no preference for normal-order over inversed-order father's songs, the strength of vocal learning may influence their approach responses to syllable-shuffled father's song.

## 4. Discussion

We found that male and female zebra finches showed differential approaching and vocal behaviour towards playbacks of the normal-order father's song versus a novel song stimulus, confirming prior results (e.g. [14,17], also see the electronic supplementary material for a discussion) and indicating recognition of the learned father's song. When we made changes to the syllable sequence, subjects differentiated between the syllable-shuffled father's and novel song with similar performance to that in the normal-order test. They were able to do this even after we had inversed the order of all syllables, thus demonstrating robust recognition of the learned father's song. In a separate test, in which we directly compared normal and inversed father's songs, males and females responded similarly to both versions of the father's song. Together, the results suggest that father's song was readily recognized, and that syllable order was not a crucial feature for recognition.

When the strength of male subjects' song imitation was taken into consideration, we found a significant correlation between differential behavioural responsiveness to the song stimuli and the similarity with the father's song. In the tests where we contrasted father's versus novel songs, males with the poorest imitations of the father's song (poor imitators) showed a stronger approach response to the father's song than subjects that had copied most of their father's song (good imitators), particularly in the test where we contrasted the inversed-order father's versus novel song. This was not owing to the good imitators losing their differential approaching response in the tests with sequence manipulations, because they demonstrated a similar response strength in the normal and inversed test. On the contrary, it seemed like the poor imitators spent more time in the father's approach zone when they heard the order-manipulated father's songs. In the test where we directly contrasted the normal-order versus inversed-order father's song, we found a significant correlation between the strength of song imitation and the approach response of males, where poor imitators tended to approach the inversed, and good imitators the normal father's song. The response of poor imitators was thus consistent with what we found in the earlier tests, but why they would respond this way remains an unanswered question. Taken together, these results suggest that in zebra finches, syllable sequence is not a crucial factor for recognition of father's song, but may affect the responsiveness of males in proportion to the strength of vocal imitation learning.

### (a) Voice recognition?

It is unlikely that our subjects recognized their father's song based on voice cues only. First, previous work has shown that voice cues were found to be weak in zebra finch vocalizations. Researchers trained a set of computational classifiers to recognize an individual's vocalizations from a pool of vocalizations by different birds. When they trained the classifiers with one type of vocalization of an individual (e.g. loud calls) and then tested recognition using different call types of the same individual, performance was poor, indicating that the classifiers could not reliably recognize any identity-bearing features, i.e. voice [18]. Second, young males that were tape-tutored with one half of a song and subsequently exposed to the unfamiliar counterpart in a playback test did not recognize the tutor [19]. Thus, it appears that zebra finches may not recognize learned song based on voice alone, but that they rely on other information present in the father's song.

### (b) The role of syllable order in zebra finches

Previous work has shed some light on the importance of syllable order for zebra finch vocal learning and song recognition. When juvenile zebra finches are tutored with a variable song sequence, i.e. five syllables presented in all possible orders an equal number of times, juveniles develop a stable, single-sequence song themselves [7]. When raised with their fathers in a single-family, laboratory cage, juveniles imitate not only the spectral features, but also the sequential pattern of the father's song (e.g. [20]). There does not seem to be any difference in sequence sensitivity between juveniles and adults [9], so if juveniles can imitate their father's song with sequential precision and sing it throughout their life with the same sequential precision, this might suggest that also adults strongly code and use this information.

Indeed, it seems that zebra finches are able to *perceive* a variation in syllable order [8,9,21]. However, psychoacoustic experiments suggest that zebra finches are extremely sensitive to the spectral details of individual syllables and rely less on syllable sequence for recognition (e.g. [10], and see [11] for a review). During such experiments, birds are usually trained to discriminate unfamiliar song stimuli without social relevance to the subjects. Interestingly, when socially relevant stimuli (songs of males housed in the same aviary) were used, sensitivity to sequence changes improved significantly [13]. Therefore, it appears that social relevance may stimulate zebra finches to pay more attention to sequential information of song syllables.

In our study, we tested song responses in a more natural context, where we observed zebra finches' spontaneous behaviour during playbacks of the socially relevant father's song. Nevertheless, we found that syllable sequence manipulations did not severely disrupt recognition of the father's song. Therefore, our results fit well with previous work questioning the salience of sound sequence in vocal learning and in song perception as described above [10,11,13]. On the other hand, it may have been relatively easy for the birds to recognize the father's song based on the presence of a partially intact song, and so they may readily recognize their father's song even from the inversed stimulus. Thus, the fact that subjects' discrimination performance was not better in the middle or normal tests may reflect a ceiling effect. Indeed, previous studies have shown that zebra finches do not need to hear all syllables for song recognition, and birds can recognize conspecific songs in which half of the song is deleted [22]. This may be important for zebra finches, as these birds live in large, loud groups in the wild, and song may be partially masked by the song of other birds [16]. In future studies, it might be interesting to extend the analysis of behavioural responses with higher precision and finer resolution, to possibly detect more subtle differences in the behaviour of the birds reflecting the strength of recognition for the intact syllable sequence.

### (c) Is the songbird brain sensitive to sequential structure of songs?

A number of studies have demonstrated the high sensitivity of neurons in premotor brain regions to the sequential structure of the bird's own song (e.g. [23]). By contrast, higher order auditory areas in the songbird brain are involved in song perception and recognition, implicated in the auditory representation of the memorized father's song [24–26], and are probably important for auditory perception-based individual recognition [27]. Some studies comparing sequence-shuffled and normal-order song stimuli show similar neural responses in these brain regions [28,29], while others did find differential responses [30,31]. It may be that only a subset of neurons is selective for sequence or auditory contexts, just like only small subsets of neurons are selective for father's song [32].

Thus, higher order auditory areas may respond strongly to spectral features of learned songs, but less to syllable sequence, while brain regions involved in song production are more selective for syllable order. In this way, the neural substrates underpinning vocal production may be involved in song sequence learning, while the higher auditory regions underlying perception may be involved in song recognition regardless of syllable-order.

## Data accessibility

The data are provided in the electronic supplementary material [33].
